# Complications After Open Skull Base Surgery for Brain Tumors: A 26-Year Experience

**DOI:** 10.7759/cureus.50312

**Published:** 2023-12-11

**Authors:** Orlando De Jesus

**Affiliations:** 1 Neurosurgery, University of Puerto Rico, Medical Sciences Campus, San Juan, PRI

**Keywords:** mortality, morbidity, neurosurgery, surgery, tumor, complications, open, skull base

## Abstract

Introduction: Open skull base surgery carries the risk of significant complications. It is important to inform patients and family members clearly of the details of these complications. This study aimed to present the numerous complications encountered with open skull base surgery for brain tumors. This report analyzed the complications experienced by patients treated with open skull base surgery by a single skull base surgeon at a single tertiary center over 26 years.

Methods: A retrospective study was performed using the University of Puerto Rico Neurosurgery database to identify patients who were managed using open skull base procedures from 1995 to 2020. The neurosurgical database for each patient had been prospectively recorded. Patients with skull base tumors under the author's care during the study period were included in the investigation. Exclusions include patients with non-tumoral conditions and non-skull base tumors and those operated using a microscopic transsphenoidal route. Patients who experienced an intraoperative or postoperative complication within 30 days of the surgery were further analyzed.

Results: In the cohort, 141 patients with brain tumors underwent open skull base surgery. The cohort had a median age of 48 (range 3-79). It consisted of 101 (71.6%) females and 40 (28.4%) males, with a female-to-male ratio of 2.5:1. The approach most frequently used was an orbitofrontal craniotomy (with or without zygomatic osteotomy) in 79 patients (56%). A petrosal approach was utilized in 26 patients (18%). Forty-six patients (33%) developed an intraoperative or postoperative complication. Twenty-four percent of the complications occurred in the 30-day postoperative period. Five patients had more than one complication. The median age of the patients who experienced a complication was 44.5 (range 22-79), with a female-to-male ratio similar to the entire cohort. Cranial nerve injury was the most frequent complication. Worsening or loss of vision in the affected eye occurred in 5.7% of the patients. A cerebrospinal fluid leak occurred in 2% of the patients. Six patients died, two of them after a massive myocardial infarction.

Conclusions: The results of this study showed that approximately one-third of the patients undergoing open skull base surgery can develop a complication. The most frequent complication was injury to a cranial nerve. A large number of complications occurred intraoperatively. The majority of the complications in patients with tumors in the posterior fossa were associated with injury to a cranial nerve. At the middle fossa, damage to the optic nerves is a noteworthy complication. Complications at the anterior fossa involved worsening of vision or myocardial infarction. Less aggressive surgery near the cavernous sinus and the petroclival region may reduce complications. Understanding the complications can help counsel patients and family members.

## Introduction

The skull base region is complex, composed of different types of tissues, and in intimate contact with many vital structures. It contains multiple compartments, divided anatomically into the anterior, middle, and posterior fossa. Most skull base surgery is currently performed using endoscopic approaches, especially at the anterior cranial fossa [1]. Endoscopic surgery is predominantly used for relatively earlier disease stages with limited skull base invasion [1]. However, open skull base approaches remain a viable option for complex and extensive skull base tumors [1-4]. Combined craniofacial resection represents the most effective approach for skull base malignancies despite the evolution and advances of endonasal endoscopic techniques [1,2,4-6]. Endoscopic and open skull base surgery aims to perform a radical resection with clear margins while limiting the complication rate [1,4]. Effective management of skull base pathology requires the involvement of a multidisciplinary team that minimizes mortality and complications [1,3,7,8]. This multidisciplinary team includes neurosurgeons, otolaryngologists, plastic surgeons, ophthalmologists, maxillofacial surgeons, anesthesiologists, pathologists, neuroradiologists, head and neck oncologists, radiation oncologists, nurses, physiotherapists, speech therapists, nutritionists, psychologists, and social workers [7].

Many technical improvements and modifications have been achieved for open skull base surgery; however, it still carries the risk of substantial perioperative complications. Complications are typically classified based on the body region affected. They are generally classified as wound, intracranial, ocular, and systemic complications [1,4,6,9]. This study aimed to present the complications encountered with open skull base surgery by a single surgeon at a tertiary center over 26 years.

## Materials and methods

A retrospective study was performed using the University of Puerto Rico Neurosurgery database to identify patients who were managed using open skull base procedures from 1995 to 2020. The neurosurgical database for each patient had been prospectively recorded, including all the complications. Patients with brain tumors under the author's care and managed using open skull base procedures from 1995 to 2020 were included in the study. Exclusions include patients with non-tumoral conditions and non-skull base tumors and those operated using a microscopic transsphenoidal route. Patients undergoing a two-stage surgery were entered once. Patients requiring reoperation for a complication were analyzed only for the original tumor surgery. The study's sample size was 141, which relied on the number of patients with skull base tumors operated by the author during the study period.

Those patients included in the study were entered into a worksheet without any identifiers for further analysis. The variables examined for each patient include gender, age, tumor histology (benign or malignant), prior surgery, prior radiotherapy, prior chemotherapy, intracranial extension, surgical approach, and the type of dural skull base reconstruction. Patients who experienced an intraoperative or postoperative complication within 30 days of the surgery were further analyzed. Complications were classified into four main categories: wound, intracranial, ocular, and systemic. Wound complications include infection, dehiscence, necrosis, and fistulas. Intracranial complications include cerebrospinal fluid (CSF) leak, meningitis, encephalitis, cerebral edema, abscess, pneumocephalus, hematoma, stroke, cranial nerve injury, and seizures. Ocular complications include loss of vision, hematoma, corneal abrasion, ectropion, enophthalmos, periorbital cellulitis, and epiphora. Systemic complications may originate or involve the cardiovascular, pulmonary, renal, endocrine, and metabolic systems. Complications were scrutinized based on the topography of the skull base region (anterior, middle, and posterior) and the extent of resection. The surgical experience was divided into two 13-year periods to assess the complication rates. Descriptive statistics were used to report frequency and median values. This study was exempt from review by the Institutional Review Board of the University of Puerto Rico Medical Sciences Campus.

## Results

Among the patients operated on for brain tumors from 1995 until 2020, 141 underwent open skull base surgery (Table 1). The median age of the patients was 48 years (range 3-79; standard deviation 15.54). The cohort consisted of 101 (71.6%) females and 40 (28.4%) males, with a female-to-male ratio of 2.5:1. Histopathological examination showed that meningioma (59%) was the most frequent tumor. Malignant tumors were present in 14% of the patients. Two patients (1.4%) underwent prior surgery by another surgeon. None of the patients had previous radiotherapy or chemotherapy treatment. The surgical approach most frequently utilized was an orbitofrontal craniotomy (with or without zygomatic osteotomy) in 79 patients (56%). A petrosal approach was used in 26 patients (18%). A basal unilateral or bilateral frontal approach was used in 15 patients (11%). An extended posterior fossa craniotomy that included limited mastoidectomy and skeletonization of the sigmoid sinus was used in 14 patients (10%). Craniofacial, extended subtemporal, and extreme lateral transcondylar approaches were employed in 5% of the patients. A primary dural closure was performed in 54% of the patients. Autologous dural graft or dural substitute was utilized in 46% of the patients. No vascularized skin flaps were used. In three patients (2%), the tumor occupied an extradural location without macroscopic evidence of dural invasion. In these three patients, the dura was not opened. Reoperations for recurrent disease were performed in 10 patients (7%), an average of 36 months after the initial surgery.

**Table 1:** Demographics of 141 patients with an open skull base surgery for a brain tumor

Variable	N (%)
Age (years) (median)	48 (range 3-79)
Male	40 (28.4%)
Female	101 (71.6%)
Malignant histology	20 (14%)
Benign histology	121 (86%)
Orbitofrontal approach	79 (56%)
Petrosal approach	26 (18%)
Basal frontal approach	15 (11%)
Extended posterior fossa approach	14 (10%)
Extended subtemporal approach	5 (3%)
Craniofacial approach	1 (1%)
Transcondylar approach	1 (1%)
Primary dural closure	74 (54%)
Autologous/dural substitute	64 (46%)

Forty-six patients (33%) developed an intraoperative or postoperative complication (Table 2). If a cranial nerve injury or brain injury occurred during the surgical procedure, the complication was considered to occur intraoperatively. In 76% of the patients, the complication arose intraoperatively, while 24% occurred postoperatively. Intraoperative complications included cranial nerve injury, visual worsening, intracerebral hematoma, arterial tear, venous thrombosis, and hemiparesis. Postoperative 30-day complications included diabetes insipidus, CSF leak, myocardial infarction, seizure, and unknown cause of death at home. Five patients (3.5%) presented more than one complication. The median age of the patients who developed a complication was 44.5 (range 22-79; standard deviation 13.00). There were 33 (71.7%) females and 13 (28.3%) males among the patients who sustained a complication, with a female-to-male ratio of 2.5:1, similar to the operated cohort. The approach most frequently associated with a complication was the orbitofrontal craniotomy; however, it was used in more than half of the patients. Cranial nerve injury (13%), excluding the optic nerve, was the most frequent complication. These include the oculomotor, trochlear, abducens, trigeminal, facial, cochlear, glossopharyngeal, and vagus nerves. Worsening or loss of vision in the affected eye occurred in 5.7% of the patients. A CSF leak occurred in three patients (2%). Two patients had transpetrosal surgery, and the other had a craniofacial surgery, all leaking through the wound. The leaks resolved with a lumbar drain in two patients and a ventriculoperitoneal shunt in the other. An intracerebral hematoma complication occurred in four patients. One patient with a frontal intracerebral hematoma was reoperated to remove the hematoma. One patient with a midbrain hematoma required a ventriculostomy due to the development of obstructive hydrocephalus. On the third patient, a small intraparenchymal hematoma was observed. The fourth patient suffered a massive subarachnoid hemorrhage two days after removing a diaphragm sella meningioma and expired a few hours later. This last patient probably bled from a partially coagulated artery near the tumor bed. Before this incident, no perioperative complications had occurred.

**Table 2:** Complications identified in 46 patients after an open skull base surgery for a brain tumor

Complication	N (%)
Cranial nerve injury	19 (13.5%)
Worsening or visual loss	8 (5.7%)
Intracerebral hematoma	4 (3%)
Diabetes insipidus	4 (3%)
Cerebrospinal fluid leak	3 (2%)
Arterial tear/thrombosis	2 (1.4%)
Myocardial infarction	2 (1.4%)
Hemiparesis	1 (0.7%)
Venous thrombosis	1 (0.7%)
Seizure	1 (0.7%)
Death at home (unknown cause)	1 (0.7%)

When the topography of the skull base region was analyzed for complications, 54% of the complications occurred with tumors located in the middle fossa, 37% in the posterior fossa, and 9% in the anterior fossa. The majority of the middle fossa complications consisted of worsening of vision (24%) or cranial nerve injuries (24%). Additional complications for tumors in the middle fossa include diabetes insipidus (12%), intracerebral hematoma (8%), CSF leak (4%), venous thrombosis (4%), arterial thrombosis (4%), and seizure (4%). Seventy-six percent of the complications at the posterior fossa were secondary to a cranial nerve injury. Other complications in the posterior fossa included CSF leak (6%), arterial tear (6%), intracerebral hematoma (6%), and hemiparesis (6%). At the anterior fossa, 50% of the complications involved worsening of vision, and 50% were associated with an event of myocardial infarction.

The extent of resection was evaluated in those patients who developed a complication. A subtotal resection was performed in 20 (43%) patients, while a gross total resection was achieved in 26 (57%) patients. The surgical experience was divided into two 13-year periods to assess the complication rates. The early period comprised 1995 until 2007, and the late period included 2008 until 2020. Sixty-seven percent of the complications occurred in the earlier period, while 33% occurred in the late period.

Six patients (4.2%) died in the postoperative period. Two of them suffered a massive myocardial infarction within one to three days postoperative. No perioperative complications were documented in these two patients before the sudden death occurred. One patient died suddenly three days after discharge without the identification of any postoperative complications. No autopsy was performed on this patient with a malignant tumor diagnosis. In three of the patients (50%), a specific complication (arterial injury or venous thrombosis) was directly associated with the death.

## Discussion

Open skull base approaches are still used for complex skull base tumors. These procedures carry significant morbidity as they are traditionally reserved for advanced and extensive cases. The literature cites a complication rate of 28-47% [2,5-14]. Mortality can range between 1.0% and 4.7% [6,8-10,14-17]. In the current study, the complication rate was 33%, with a mortality of 4.2%, which is in accordance with previous literature reports. Some recent studies have utilized the American College of Surgeons National Surgical Quality Improvement Program (NSQIP) database to analyze the complications; however, this database does not record surgical-specific complications for each procedure, like cranial nerve injury, CSF leaks, postoperative hematomas, or visual deficits [17-18]. Studies using the NSQIP database focus more on systemic complications, hospital length of stay, unplanned intubations, and ventilator dependence, therefore underestimating the overall complication rate [17-18].

Miller et al. reported that age was not associated with an increased risk of complications [2]. However, Gil et al. and Singh et al. demonstrated that advanced age constituted a significant risk for developing postoperative complications and mortality [9,12]. Several studies have indicated that tumor malignancy is not associated with an increased risk of complications [2,8,11]. However, Ringel et al. found that malignant pathology predicted delayed (>30-day post-surgery) complications [7]. Stephenson et al. showed that in patients over 70 years, increasing age or malignant disease did not influence the complication rate [19]. In the current study, the number of malignant tumors was low; thus, an association with the complication rate cannot be established. Several authors have documented that medical comorbidities significantly predict postoperative complications and mortality [6,12,17,20]. Ganly et al. demonstrated that patients were twice as likely to die if they had a major medical comorbidity [6]. However, Sakashita et al. found that medical comorbidities were not associated with an increased risk of complications [11]. Kuan et al. indicated that a higher American Society of Anesthesiologists physical status class significantly predicts complications and mortality [13].

Prior radiation therapy had been significantly associated with higher complication rates [3,6,7]. However, Deschler et al. noted no association between a higher complication rate and previous chemotherapy, radiation, or surgery [8]. Kraus et al. and Miller et al. also demonstrated no association with prior radiation [10,21]. Sakashita et al. noted that the complication rate for cases with prior chemotherapy, radiation, surgery, or dural involvement with resection is significantly increased [11]. However, Kraus et al. and Miller et al. showed that prior surgery did not influence the complication rate [10,15,21].

Infection is the most frequent postoperative complication encountered with anterior craniofacial surgery [2,5,6,8]. Prior radiation therapy increases the risk of infection in this type of surgery due to poor tissue vascularity [3,6]. Using a broad-spectrum antibiotic regime decreases the complication rate for anterior craniofacial resection by reducing the incidence of wound complications [3,4,7,9,15]. Miller et al. found that current open skull base surgery wound complications are comparable to those preceding the endoscopic era [2]. The authors proposed this occurred because the open approach had been reserved for the most extensive and complex lesions traditionally associated with increased morbidity. Postoperative complications are reported to occur more frequently after craniofacial resection and infratemporal fossa approaches than orbitozygomatic and transfrontal approaches [2]. However, the approach most often associated with a complication in the current study was the orbitofrontal craniotomy. This observation can be justified as most tumors were meningiomas, with a minority of malignant cases and a single craniofacial resection case. 

Brain invasion/extension significantly predicts postoperative complications [6,7]. Dural invasion also increases the risks of complications [6,10]. Vascularized tissue transfer has minimized the complication and mortality rate [3,7]. Vascularized non-irradiated tissue should be used for the reconstruction to prevent wound complications [3]. However, others believe that the use of flap reconstruction increases the complication rate [2,8,20]. Ganly et al. showed that using vascularized tissue for reconstruction compared to non-vascularized tissue did not reduce overall complications [6]. Also, there was no difference in the complication rate when comparing pedicled flaps to free flaps and non-vascularized tissue [6]. In the current study, vascularized flaps were not used as craniofacial resection comprised a minimal number of cases. Designing scalp flaps considering the regional blood supply with adequate reconstruction of dural and bone defects helps prevent cosmetic defects and decrease the rate of significant complications [1,3,4].

Vascular complications are potentially the most severe complications following skull base surgery [3]. These can include carotid rupture and arterial embolism secondary to dissection and manipulation of the carotid arteries [3]. Thrombosis or occlusion of the draining veins or venous sinus can produce significant cerebral edema and cerebral venous infarction [3]. In the current series, one death occurred secondary to a vertebral artery tear. The tear in the artery was sutured; however, it caused a large brainstem stroke. Another patient developed severe brain edema after the temporal draining veins were coagulated. Postoperative intracerebral hematomas need reoperation in most cases. In this series, an intracerebral hematoma complication occurred in four patients. One patient required reoperation for the removal of the intracerebral hematoma.

Open skull base surgery for petroclival meningioma produces significant new postoperative cranial nerve deficits ranging from 34% to 44%, most often weakness of the facial nerve and extraocular muscles' cranial nerves [14,22]. CSF leak occurs less frequently with petroclival meningioma surgery than with anterior craniofacial skull base surgery, as dural defects are smaller and more manageable to reconstruct. We had an overall 13% of new cranial deficits in the entire cohort of patients; however, new postoperative cranial nerve deficits occurred in 33% of the patients with petroclival meningiomas. It has been shown that reoperation for petroclival meningioma tumors has a higher rate of overall complications [22,23]. Early rehabilitation of residual deficits is essential to the skull base practice [16,22].

Previous authors have observed that open skull base surgery perioperative death can be associated with myocardial infarction [8,10,13,19]. In the current study, two deaths were directly attributed to myocardial infarction. Ocular complications can produce significant disability for the patients. In this series, there was a 5.7% worsening or loss of vision in the affected eye. In half of these patients, the preoperative visual acuity of the involved eye was worse than 20/400. Two patients with normal vision lost sight in the ipsilateral eye due to excessive dural coagulation of the parasellar tumor bed. Seventy-five percent of the patients with a postoperative visual complication were operated in the first 10 years of the series. During the 26-year surgical experience, the author demonstrated an evolution of the surgical philosophy without abandoning open skull base surgery, leaning toward less aggressive surgery with more subtotal resections to reduce the complications. When the surgical experience was divided into two equal periods, two-thirds of the complications occurred early, while only one-third occurred late. Many extraocular cranial nerve injuries or trigeminal nerve damage happened at the beginning of the series, where aggressive cavernous sinus or petroclival surgery was attempted to remove the tumor completely. Now, subtotal tumor resections are more frequently used. Residual tumors with benign histology are usually observed, reserving radiosurgery for those that show rapid progressive growth. Although the complication rate reduction could have arisen from surgeons' expertise acquired with years in practice, it was probably due to the role of evolving technology and the modification in surgical philosophy considering less aggressive surgery with more subtotal resections.

This study contained several limitations. The study's design was retrospective with inherent bias; however, it included a consecutive series with prospectively recorded data. Additionally, operations were performed by one surgeon at a single institution, which may limit the applicability of the results to other institutions. As only one surgeon was involved, consistency in surgical technique was maintained. However, the caseload was not restricted to skull base surgery, limiting the number of yearly cases managed. Some minor systemic medical complications may have been missed as they were not included in the patient's database. This study did not analyze the influence of smoking, medical comorbidities, or length of hospitalization on the complication rate. Clinical outcomes were not evaluated in this study. Lastly, the study period extended across 26 years, which may limit comparison with older or more recent series. 

## Conclusions

This study showed that approximately one-third of the patients undergoing open skull base surgery could develop a complication. Overall, the most frequent complication was injury to a cranial nerve. A large number of complications occurred intraoperatively. The majority of the complications in patients with tumors in the posterior fossa were associated with injury to a cranial nerve. At the middle fossa, damage to the optic nerves is a noteworthy complication. Complications at the anterior fossa involved worsening of vision or myocardial infarction. Less aggressive surgery near the cavernous sinus and the petroclival region can reduce complications.
